# The Longevity-Frailty Hypothesis: Evidence from COVID-19 Death Rates in Europe

**DOI:** 10.3390/ijerph19042434

**Published:** 2022-02-20

**Authors:** Sammy Zahran, Levi Altringer, Ashok Prasad

**Affiliations:** 1 Department of Economics, Colorado State University, Fort Collins, CO 80523, USA; levi.altringer@colostate.edu; 2 Department of Epidemiology, Colorado School of Public Health, Fort Collins, CO 80045, USA; 3 Department of Chemical and Biological Engineering, Colorado State University, Fort Collins, CO 80521, USA; ashok.prasad@colostate.edu

**Keywords:** COVID-19, frailty, longevity

## Abstract

By the end of spring (31 May), the COVID-19 death rate was remarkably unevenly distributed across the countries in Europe. While the risk of COVID-19 mortality is known to increase with age, age-specific COVID-19 death rates across Europe were similarly unevenly distributed. To explain these mortality distributions, we present a simple model where more favorable survival environments promote longevity and the accumulation of health frailty among the elderly while less favorable survival environments induce a mortality selection process that results in lower health frailty. Because the age-related conditions of frailty render the elderly less resistant to SARS-CoV-2, pre-existing survival environments may be non-obviously positively related to the COVID-19 death rate. To quantify the survival environment parameter of our model, we leveraged historic cohort- and period-based age-specific probabilities of death and life expectancies at age 65 across Europe. All variables are significantly correlated with indicators of frailty like elderly dependence on others for personal and household care for a subset of European countries. With respect to COVID-19 death rates, we find significant positive relationships between our survival indicators and COVID-19 death rates across Europe, a result that is robust to statistical control for the capacity of a healthcare system to treat and survive infected persons, the timing and stringency of non-pharmaceutical interventions, population density, age structure, case rates and the volume of inbound international travelers, among other factors. To address possible concerns over reporting heterogeneity across countries, we show that results are robust to the substitution of our response variable for a measure of cumulative excess mortality. Also consistent with the intuition of our model, we also show a strong negative association between age-specific COVID-19 death rates and pre-existing all-cause age-specific mortality rates for a subset of European countries. Overall, results support the notion that variation in pre-existing frailty, resulting from heterogeneous survival environments, partially accounts for striking differences in COVID-19 death during the first wave of the pandemic.

## 1. Introduction

**Figure 1:**
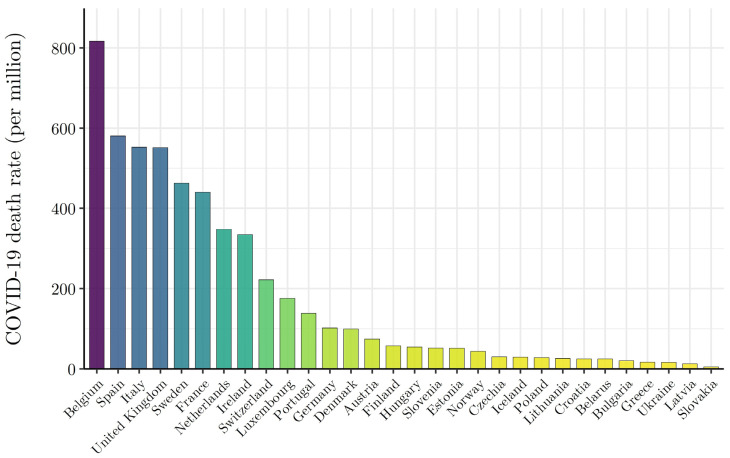
Uneven Distribution of Wave I (ending 31 May 2020) COVID-19 mortality risk across the countries of Europe. NOTE: The countries of Europe are arranged in descending order by reported COVID-19 deaths per million, as of 31 May 2020. COVID-19 death data by country are from Johns Hopkins Coronavirus Resource Center (JHU CSSE) (https://github.com/CSSEGISandData/COVID-19 Unified-Dataset (accessed on 15 June 2020)).

At the end of spring (31 May), the COVID-19 death rate (deaths over population) was strikingly unevenly distributed across Europe (Figure 1). Among the hardest hit countries in the first wave of the pandemic were Belgium, Spain, Italy, and the United Kingdom with COVID-19 death rates of 816, 580, 553, and 552 deaths per 1 million, respectively. As of 31 May 2020, the United Kingdom’s COVID-19 death rate exceeded the similarly developed economy of Germany (102 per million) by more than a factor of 5. Few epidemiological indicators distinguish the countries of Europe by such an extent.

**Figure 2:**
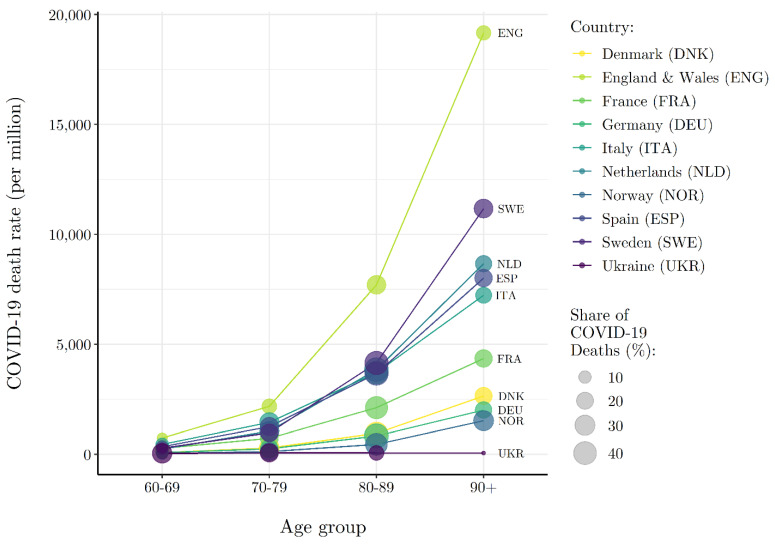
Heterogeneous Wave I (ending 31 May 2020) COVID-19 mortality risk by age group across select European countries. NOTE: COVID-19 death and population data (closest to 31 May 2020) by age and country are from National Institute for Demographic Studies (INED) (https://dccovid.site.ined.fr/en/data/ (accessed on 15 June 2020)).

Because the risk of COVID-19 death is significantly higher among the elderly, one might assume that variation in the COVID-19 death rate across Europe in this period reflects differences in age structure [1,2]. Consider Figure 2 that plots age-specific COVID-19 death rates (as of 31 May 2020) for nine European countries with readily available data. The size of each point plotted in Figure 2 reflects the share of total reported COVID-19 deaths that can be attributed to each age group within a country. Most striking is that the cross-national variation observed in Figure 1 remains. England’s age-specific death rates are roughly five times that of Germany’s across all age groups. The within-age-group variation in COVID-19 death rates across countries implies that the age structure of the population cannot account for observed differences in COVID-19 mortality risk between European countries. In fact, the percentage of the population ≥ 65 (*r* = 0.134, *p* = 0.480) and ≥75 (*r* = 0.186, *p* = 0.325) years of age is statistically uncorrelated with the COVID-19 death rate during this phase of the pandemic.

While the percent of elderly is statistically independent of the COVID-19 death rate, the risk of death by COVID-19 is manifestly related to age. Except for Ukraine, across the countries in Figure 2, persons 70 years of age and older account for more than 80% of all reported COVID-19 deaths. A theory of the puzzling distribution of the COVID-19 death rate across Europe at the end of spring must therefore account for the age-related nature of COVID-19 death risk.

Frailty is a notion used to describe a range of age-related conditions that render the elderly less resistant to health shocks [3,4,5,6]. Several clinically validated measures of frailty such as muscle weakness, fatigue, low physical activity, poor balance, visual impairment, and cognitive impairment that increase elderly dependence on others are correlated with age. The accumulation of frailty in a society may be non-obviously positively related to the survival environment [7,8]. Societies characterized by higher pre-existing rates of elderly survival and consequent frailty accumulation may be more susceptible to higher rates of COVID-19 death, at least initially before the arrival of more effective treatment modalities.

Consider two similarly economically developed countries with varying rates of survival to age 75. Suppose that one country has high and the other a low survival environment with respect to all causes of death. Persons surviving to 75 in the low survival environment are more positively selected on the underlying ability to persist from 1 year to the next—i.e., longevity. Given positive mortality selection, elderly persons over the age of 75 in the low survival country may be more likely to withstand adverse health shocks than similarly aged persons in the high survival country because of lower underlying frailty. Similarly, persons surviving to 75 in a high survival environment are more negatively selected on the underlying ability to persist, rendering such persons less likely to withstand adverse health shocks than similarly aged persons in a low survival environment because of higher underlying frailty.

In this paper we develop a simple and highly stylized model of longevity and accumulated health frailty that captures the intuition of positive and negative mortality above more formally. Each country begins with an identical underlying distribution of health frailty among the population. Then, heterogeneous survival environments across countries induce mortality selection processes that generate varying amounts of accumulated health frailty in subsequent periods. Specifically, more favorable survival environments promote longevity and a larger amount of accumulated health frailty, while less favorable survival environments induce a relatively strict mortality selection process that results in a lower amount of accumulated health frailty among the elderly. With respect to an exogenous health shock that disproportionately targets the relatively health frail, like SARS-CoV-2, this simple model implies that countries with more favorable pre-existing survival environments will have larger susceptible (health frail) populations and, therefore, higher COVID-19 death rates.

To measure a survival environment, we collected data on pre-existing all-cause age-specific death rates (measured in both period and cohort-specific terms), data on life expectancy at age 65, and developed an index of survival that is integrative of these measures. All measures of the survival environment are highly correlated with more explicit indicators of elderly frailty, including percent of disabled persons ≥ 65 years that need the assistance of others, percent of persons ≥ 65 years that need help with personal care activities, and percent of persons ≥ 65 years that need help with household activities. Across measures of the survival environment, we show that a more favorable pre-existing survival environment is strongly positively associated with the COVID-19 death rate across Europe. These results are robust to statistical control for competing theories of COVID-19 prevalence and mortality, including the capacity of a healthcare system to manage and survive infected persons, the timing and stringency of non-pharmaceutical interventions, population density, age structure, and the volume of inbound international travelers. Results are also robust to the substitution of our response variable—the COVID-19 death rate—with a measure of cumulative excess mortality that addresses possible concerns of reporting heterogeneity across Europe. Finally, for a subset of European countries with readily available data, and compatible with our *longevity-frailty model*, we show a strong negative association between age-specific COVID-19 death rates and pre-existing all-cause age-specific mortality rates.

In the next section we describe our theoretical model, outlining the basic relationship between a country’s survival environment and the expected accumulation of health frailty among the elderly. In Section 3 we detail data sources, variable operations, and statistical models to test our model. In Section 4 we present results, including a series of robustness tests. In Section 5 we conclude with a summary of key findings and how our simple model is consistent with other facts of the first wave of the COVID-19 pandemic in Europe.

## 2. Model

Consider a very simple model of survival with two periods, t=(1,2). In period t=1, a country (j) begins with a representative population of individuals (i) who are each in possession of a level of health frailty ($f_{i}$). Health frailty ranges from 0 (least frail) to 1 (most frail) and, for simplicity and illustration of logic, is assumed to be distributed normally across the population with the restriction that Pr[0≤f≤1]=1 since f∈[0,1]. The assumed distribution of health frailty across the population is inconsequential for the comparative statics of this simple model.

The likelihood that an individual survives to period t=2 is determined by a Bernoulli trial. Specifically, individual i in country j survives to period t=2 with probability $p\left(f_{i};\delta_{j}\right)$ or, equivalently, dies in period *t* = 1 with probability $1-p\left(f_{i};\delta_{j}\right)$. The survival probability function p(f;δ), defined over the domain of health frailty, and dropping the subscripts for simplicity, takes the following functional form

(1) $$p\left(f;\delta \right)=1-f^{\delta }$$

where δ>1. Taking the first and second derivative of the survival probability function with respect to health frailty shows that the probability of survival increasingly deteriorates with health frailty

(2) $$\frac{\partial p}{\partial f}=-\delta \;f^{\delta -1}<0$$

(3) $$\frac{\partial^{2}p}{\partial f^{2}}=-\delta \left(\delta -1\right)f^{\delta -2}<0$$

with certain survival for the least frail—p(0;δ)=1—and certain death for most frail—p(1;δ)=0. For a given level of health frailty in the range (0, 1), a more favorable survival environment increases an individual’s likelihood of surviving into older age. Specifically,

(4) $$\frac{\partial p}{\partial \delta }=-\ln\left(f\right)f^{\delta }>0$$

so that larger values of δ reflect a more favorable survival environment.

Given the survival probability function, we can derive the expected accumulation of health frailty in period t=2. Let $N_{f}^{1}$ denote the number of individuals in period t=1 with health frailty f. Then the expected number of persons with health frailty f≥h that survive to period t=2 is given by

(5) $$E\left[N_{f\geq h}^{2}\right]=\int_{h}^{1}N_{f}^{1}p\left(f;\delta \right)df=\;\int_{h}^{1}N_{f}^{1}\left(1-f^{\delta }\right)df$$

and the expected number of relatively frail individuals (f≥h) that survive to period t=2 is increasing in δ

(6) $$\frac{\partial E\left[N_{f\geq h}^{2}\right]}{\partial \delta }=-\int_{h}^{1}N_{f}^{1}\ln\left(f\right)f^{\delta }df>0;\;\;\;\;\;h\in \left(0,1\right).$$

Thus, this simple model outlines a clear relationship between a country’s survival environment (δ) and the expected accumulation of health frailty among the elderly (period t=2). It is important to note that an individual’s health frailty is not endogenously determined in this simple model. For instance, a less favorable survival environment may positively contribute to the accumulation of health frailty through “scarring” or “weathering” effects even though the process of mortality selection negatively contributes to accumulated health frailty, or what some refer to as the “culling” effect [9]. A model that incorporates these competing effects would provide a more general theoretical understanding of accumulated health frailty that could account for within-country variation in health outcomes and/or mortality across groups—for example, the relatively high COVID-19 death rates observed for African Americans in the US [10] or the positive association between COVID-19 death risk and multiple deprivation in the UK [11].

That said, because frail persons are more susceptible to a range of age-related conditions that render them less resistant to health shocks or sudden changes to the survival environment like SARS-CoV-2, we predict that higher survival environments are positively associated with higher COVID-19 death rates. While we do not explicitly model the determination of δ, this environmental parameter is a function of several factors that contribute to the longevity of individuals across a health frailty distribution—e.g., income, nutrition, public health, vaccination, medical treatments, and social welfare policy—and, therefore, δ is likely to vary within countries over time [12]. These factors may operate on populations in period-specific ways or cumulatively on a specific cohort over the life-course. For example, the period-based influenza hypothesis argues that COVID-19 death rates are higher in Sweden than neighboring Nordic countries because Sweden experienced remarkably lower death rates from flu over the last seasons [13]. The period-specific attenuation of influenza mortality produced relatively higher rates of frailty that rendered the elderly in Sweden more susceptible to COVID-19 death. In the next section, we develop and describe various indicators of the survival environment used to test our model.

## 3. Data and Methods

### 3.1. Survival Environment Indicators

Cohort Survival. To obtain a cohort-based measure of survival environments across the countries of Europe we employ cohort death rate data from the Human Mortality Database (HMD). HMD data were accessed at https://www.mortality.org/ (accessed 15 June 2020). First, we subset the HMD data for countries in our sample to all cohorts born between 1910 and 1960—i.e., data for individuals at least 60 years of age in the year 2020—and collect $m_{x}$, the observed death rate at age x. This generates different amounts of data for each country-cohort group. For instance, for the cohort born in 1920, data may be available for the age range of 0–106 years. For the cohort born in 1960, however, death rate data will only be available for the 0–56 years age range. Further, there is missing death rate data for some cohort-age observations for some countries. For the sake of comparability across countries, we subset the cohort $m_{x}$ data to all cohort-age observations that are available for all countries in our sample. Thus, the cohort-based measure of survival is generated using an age-truncated sample of data—e.g., x = 23 to x = 102.

To calculate $q_{x}$ for each country-cohort-age group, which is defined as the probability that a person of age x will die within one year, we perform the following life table calculation

(7) qx=mx(1+αxmx)=mx(1+0.5mx)

under the imposed assumption (shown above) that $\alpha_{x}\;$= 0.5 for all age groups, which is consistent with the HMD Methods Protocols (pp. 36–37) [14]. Thus, for each country-cohort-age observation we are able obtain $1-q_{x}$ which is the probability that a person of age x will survive to the following year. To get an average measure of the survival environment across cohorts for each country, we perform a LOESS regression of $1-q_{x}$ on age to get $1-q_{x}$ values.

To get an overall measure of the cohort-based survival environment across all age groups by country, we then perform the following calculation

(8) $$\overset{102}{\underset{x=23}{\sum }}1-{\tilde{q}}_{x}$$

which is a discrete measure of the area under the $1-q_{x}$ curve. For reference, across the 28 European countries in our sample for which cohort data are readily available—Germany and Croatia excluded—the minimum area calculated $1-q_{x}$ is 73.05 (Bulgaria), the maximum area calculated is 75.41 (France), and the mean and median areas are 74.62 and 74.37, respectively.

Period Survival. We employ the most recently available all-cause age-specific death rate data from HMD life tables—2016 for all countries except for Ukraine where the most recently available data are 2013—to obtain our first period-based measure of the survival environment for the countries of Europe. Specifically, for each country-age group—0–110 years for each European country—we collect $q_{x}$, which, again, is defined as the probability that a person of age *x* will die within 1 year. Then, transforming this measure to $1-q_{x}$ we obtain the probability that a person of age x will survive to the following year. Given that HMD measures terminate at 110 years of age, we impose the restriction that $1-q_{110}=0$ for all countries.

Like our cohort-based measure of survival environment, we perform the area under the curve calculation

(9) $$\overset{110}{\underset{x=0}{\sum }}1-q_{x}$$

to get an overall measure of the survival environment across all age groups, by country. Across the 30 European countries in our sample, the minimum area calculated is 99.84 (Bulgaria), the maximum area calculated is 102.43 (France), and the mean and median areas are 101.42 and 101.55, respectively. In addition to the period-based measure presented above, we also employ the most recently available life table measure *of* *life expectancy at 65 years of age* as a period-based measure of survival environment. This is obtained directly through the HMD life tables.

Survival Index. Our three indicators of survival (described above) are all highly correlated. Thus, using Principal Component Analysis (PCA), we combine our cohort- and period-based measures of survival environment into one integrative index. Our results indicate that roughly 94% of the variation observed across these three measures is captured through the first principal component. The standardized predicted PCA score for each country in our sample serves as an additional indicator of survival to be employed in analyses of COVID-19 death risk across the countries of Europe.

Validation of Survival Environment Indicators. Our simple model outlines a clear relationship between a country’s survival environment and the accumulation of health frailty among the population. Insofar as our indicators of survival are an appropriate characterization of relative differences in survival environments across the countries of Europe, it follows that they can proxy, albeit roughly, for relative differences in the accumulation of health frailty.

Using data from the European Commission Eurostat database, we test the conceptual validity of our simple model and indicators of survival. Eurostat data were accessed at https://ec.europa.eu/eurostat/data/database (accessed on 15 June 2020). Data are available for 14 of the 30 countries in our sample—see Appendix A Table A1 for the list of countries where Germany is excluded given the absence of a cohort-based indicator of survival environment. We begin by constructing an elderly care index from three separate measures of elderly frailty; percent of disabled persons ≥ 65 years that need the assistance of others, percent of persons ≥ 65 years that need help with personal care activities, and percent of persons ≥ 65 years that need help with household activities. Employing PCA, we derive an integrative index of the need for elderly care using these measures. Across these three measures that proxy for elderly frailty and the need for care, the first principal component accounts for roughly 68% of overall variation.

**Figure A1:**
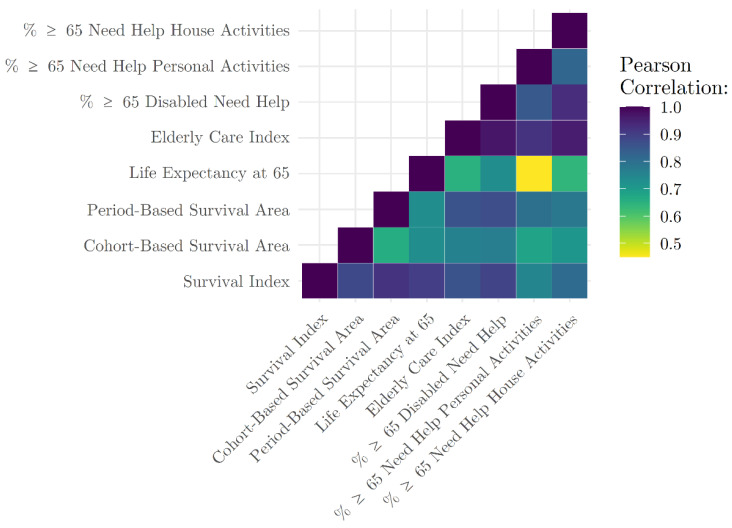
Correlation matrix for indicators of survival environment and elderly frailty. NOTE: Survival environment indicator data are derived from the Human Mortality Database (HMD) (https://www.mortality.org/ (accessed on 15 July 2020)) and elderly frailty indicator data are from European Commission Eurostat database (https://ec.europa.eu/eurostat/data/database (accessed on 15 July 2020)). The data underlying the correlations presented here can be found in Appendix A Table A1.

In Appendix A Table A1 we present scores for the three indicators of survival and the Survival Index alongside the three measures of elderly frailty and the Elderly Care Index for 13 similarly economically developed countries. The four countries that score highest according to our Survival Index—France, Spain, Italy, and the United Kingdom—also score highest on the Elderly Care Index, in the same order. Additionally, Iceland, Austria, and Finland rank in the bottom four countries across both indices. Further, in Appendix A Figure A1, we present a correlation matrix that, using the data presented in Appendix A Table A1, shows that each individual indicator of the survival environment and need for elderly care, as well as associated indices, are positively correlated with each other. We see this as an indication that our Survival Index, comprised of various indicators of the survival environment, proxy for relative differences in accumulated health frailty among the elderly across our subset European countries.

### 3.2. COVID-19 Mortality Risk

Our measure of COVID-19 mortality risk comes from the John Hopkins University Center for System Sciences and Engineering COVID-19 Unified Dataset. JHU CSSE data were accessed at https://github.com/CSSEGISandData/COVID-19 Unified-Dataset (accessed on 15 June 2020). Specifically, we combine COVID-19 death data with population data from the UN to calculate the daily cumulative COVID-19 death rate (deaths divided by population) over the period of 4 March–31 May 2020, corresponding to the end of spring and the first wave of the pandemic.

We note here that the COVID-19 death rate may be biased by testing regimes and capabilities. The issue of testing bias is particularly problematic for our analysis if COVID-19 testing regimes and capabilities are correlated with survival environments. For instance, one might expect that countries with greater COVID-19 testing capabilities are likely to be the same countries that have the healthcare infrastructure necessary to cultivate a relatively favorable survival environment. This would result in a positive relationship between the COVID-19 death rate and survival environment that is merely an illusion of testing capabilities. The standard econometric approach in this case would be to employ a fixed effects regression model, where unobserved heterogeneity in the response variable—such as testing capabilities—is controlled for through country fixed effects. However, given the time-invariant nature of our variables of interest, as well as the other control variables included in or model, we are unable to undertake this approach. Instead, we employ a second-best alternative—random effects regression model—which we discuss below. In the next section, we describe a series of control variables that account for these alternative explanations.

### 3.3. Control Variables

The control variables included in our empirical models account for alternative hypotheses of variation in COVID-19 mortality risk. These hypotheses include the timing and strength of epidemic seeding from exported cases; the capacity of healthcare systems to manage and survive infected persons; the stringency of non-pharmaceutical interventions (NPI); population density; the confirmed case rate to account for epidemic intensity; age structure; and whether a country requires mandatory Bacille Calmette-Gu’erin (BCG) vaccination, suspected to ameliorate SARS-CoV-2 infection [15].

To control for timing and strength of international seeding, we collected the most recently available data from the World Bank on the annual count of inbound international tourists (2018). The intuition being that a country with a larger number of inbound international tourists would likely have a stronger initial seeding of the virus, which would likely influence the trajectory of the pandemic in each country. Population density data are from the World Bank, capturing the count of persons per square kilometer as of 2019, proxying for risk of spread. We also include World Bank data on hospital beds per capita (2015/2016), physicians per capita (2015/2016), and nurses per capita (2015/2016) to control for the capacity of healthcare systems in each country to manage and survive infected persons. Our measure of NPI stringency comes from the Oxford COVID-19 Government Response Tracker and is collected from the John Hopkins University Center for System Sciences and Engineering COVID-19 Unified Dataset. The Oxford COVID-19 Government Response Tracker collects information on several common policy responses that governments have implemented to respond to the pandemic, such as school closures, workplace closures, cancellation of public events, and stay at home orders to name a few. We use the Stringency Index, which combines information about containment and closure policies with information about public information campaigns to generate a daily stringency score for each country that ranges from 0 to 100. Our models also control for the confirmed case rate, meant to proxy for epidemic intensity. These data are also from the John Hopkins University Center for System Sciences and Engineering COVID-19 Unified Dataset. Finally, with data from the World Bank, we include a control for age structure, captured as the percent of the population over the age of 65 years or older.

### 3.4. Statistical Models

Due to the time-invariant nature of our survival environment measures of interest, as well as the cross-sectional time-series nature of the data, we estimate versions of the following random effects model

(10) $$\ln\left(CoV_{jt}\right)=\alpha +\beta SEI_{j}+\delta X_{j}+\gamma Z_{jt}+\nu_{j}+\epsilon_{jt}$$

with

(11) $$\epsilon_{jt}=\rho_{j}\epsilon_{jt-1}+\eta_{jt}$$

where j indexes refers country, and *t* indexes refers to day. CoV is the COVID-19 death rate. SEI is the survival environment indicator of interest, which, as we have previously shown, proxies for the accumulation, or build-up, of health frailty in a country. X is a vector of time invariant control variables, including the number of inbound international tourists, hospital beds per capita, physicians per capita, nurses per capita, percent elderly, population density, the confirmed case rate, and mandatory BCG vaccination status (a), while Z represents our only time-variant control, NPI policy stringency. $\nu_{j}$ is a country-specific random effect, and $\epsilon_{jt}$ is the model disturbance term, which is assumed to follow the AR(1) process shown above—where |ρ|<1 and $\eta_{jt}$ is independently and identically distributed (*i.i.d.*) with mean 0 and variance *σ_η_*^2^. The country-specific random effect is meant to account for omitted variables like cross-national differences in comorbidities like diabetes, cardiovascular disease, as well as demographic risk factors such as socioeconomic status linked to COVID-19 mortality.

We estimate three specifications of the general model presented above. First, we include our measures of interest, the indicators of survival environment (*SEI*), as a continuous variable—the natural log of life expectancy at 65 (ln*LE*_65_), the natural log of the cohort-based survival area (ln*CS*), the natural log of the period-based survival area (ln*PS*), and the natural log of the Survival Environment Index (ln*SI*)—so that

(12) $$\ln\left(CoV_{jt}\right)=\alpha +\beta \ln\left(SEI_{j}\right)+\delta X_{j}+\gamma Z_{jt}+\nu_{j}+\epsilon_{jt}$$

with *ϵ_jt_* defined to follow the AR(1) process described by Equation (11). According to our theoretical intuition that links favorable survival environments with larger amounts of accumulated health frailty, we expect the estimated β coefficient to be positive. In other words, we expect that countries with pre-existing survival environments that are relatively more favorable will have a higher COVID-19 death rate, all else equal. Specifically, the estimated β coefficient is interpreted as elasticity. For example, in the case where the survival indicator is ln*LE*_65_, a 1% increase in life expectancy at 65 is associated with a β percent increase in the COVID-19 death rate, all else equal.

Second, we include the indicators of survival environment as a categorical variable by grouping our sample of European countries into terciles along the measure of interest so that

(13) $$\ln\left(CoV_{jt}\right)=\alpha +\beta_{1}SEI_{j}^{2nd}+\beta_{2}SEI_{j}^{3rd}+\delta X_{j}+\gamma Z_{jt}+\nu_{j}+\epsilon_{jt}$$

where ${SEI}_{j}^{2nd}\left({SEI}_{j}^{3rd}\right)$ is a dummy variable equal to 1 if country *j* is in the second (third) tercile group for the survival environment indicator of interest—e.g., life expectancy at 65—and 0 otherwise. Again, $\epsilon_{jt}$ is the model disturbance term that is assumed to follow the AR(1) process shown above—where |ρ|<1 and $\eta_{jt}$ is independently and identically distributed (*i.i.d.*) with mean 0 and variance *σ_η_*^2^. Here, the reference group is all countries that fall into the first tercile range of the measure of interest. The interpretation of the β coefficients is less straight forward than in the previous specification. The exponentiated coefficient exp(*β*_1_) is the ratio of the expected geometric mean for the second tercile group over the expected geometric mean for the first tercile group, all else equal. Thus, [exp(*β*_1_) − 1] × 100 gives the associated percentage increase, or decrease if the difference is negative, in the COVID-19 death rate for the second tercile group relative to the first tercile group, all else constant [16].

Lastly, we continue with the categorical treatment of the Survival Index (*SI*) and interact it with week fixed effects to allow the estimated relationship between *CoV* and *SI* to vary over time. Specifically,

(14) $$\ln\left(CoV_{jt}\right)=\alpha +W_{t}+\beta_{1}SI_{j}^{2nd}+\beta_{2}SI_{j}^{3rd}+\beta_{3}W_{t}\times SI_{j}^{2nd}+\beta_{4}W_{t}\times SI_{j}^{3rd}+\delta X_{j}+\gamma Z_{jt}+\nu_{j}+\epsilon_{jt}$$

where $W_{t}$ are the included week fixed-effects. In analyses that follow, we report the results of this model via post-estimation predicted margins, visually representing the divergence of COVID-19 deaths across survival environments in time.

## 4. Results

We begin with a simple assessment of the relationship between the COVID-19 death rate and our indicators of the survival environment. As expected by our model, we find strong positive correlations between the COVID-19 death rate and our indicators of the survival environment: life expectancy at 65 (*r* = 0.72, *p <* 0.001), period survival (*r* = 0.69, *p <* 0.001), cohort survival (*r* = 0.74, *p <* 0.001), and our survival index (*r* = 0.73, *p <* 0.001). In other words, higher pre-existing rates of elderly survival are associated with higher rates of COVID-19 death.

**Figure 3:**
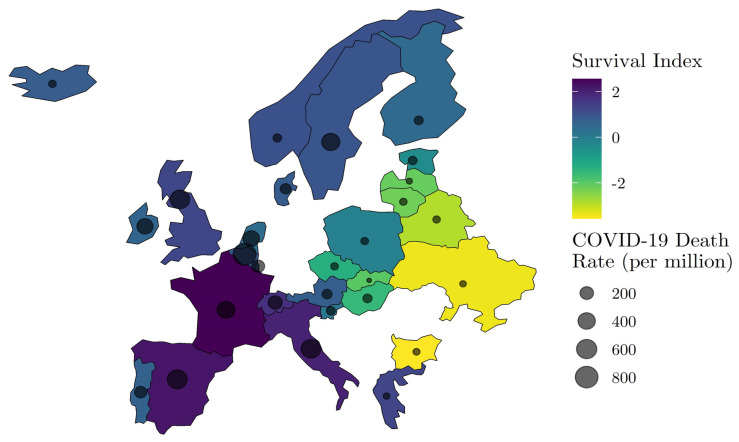
Spatial distribution of Wave I (ending 31 May 2020) COVID-19 mortality risk and relative survival environment. NOTE: COVID-19 death data by country are from Johns Hopkins Coronavirus Resource Center (JHU CSSE) (https://github.com/CSSEGISandData/COVID-19 Unified-Dataset (accessed on 15 June 2020)), and Survival Index data are derived from the Human Mortality Database (HMD) (https://www.mortality.org/ (accessed on 15 June 2020)).

Figure 3 displays the correlation spatially. Countries in yellow have the most unfavorable survival environments, while countries in purple have the most favorable, according to our survival index that integrates our three indicators. Hyper-imposed on the map are grey circles of varying size, corresponding to the observed COVID-19 death rate as of 31 May 2020. The spatial correspondence between pre-existing elderly survival and the COVID-19 death rate is evident.

Next, we test whether observed relationships between the COVID-19 death rate are robust to inclusion of variables that operationalize other candidate hypotheses, including timing and strength of epidemic seeding from exported cases, the capacity of healthcare systems to manage and survive infected persons, the stringency of non-pharmaceutical interventions (NPI), age structure, population density, epidemic intensity as measured by the confirmed case rate, and whether a country requires mandatory Bacille Calmette-Guerin (BCG) vaccination. Importantly, our statistical models include a country random-effect and week fixed-effects to account for unobserved heterogeneity by place and time.

Table 1 reports regression coefficients from our random effects regression model (Equation (12)) for all measures of the survival environment with and without control variables. The estimate of *β*, corresponding to our measures of the survival environment, is expected to be positive, indicating that higher pre-existing rates of survival among the elderly are associated with higher COVID-19 death rates. The necessary assumption for the identification of *β* is *E*[*v_j_*|SE*_j_*|] = 0, with a similar condition being required for the identification of the coefficients on the other covariates. In other words, *v_j_* is assumed to be the realization of an *i.i.d.* process with mean 0 and variance *σ_η_*^2^.

As expected, all coefficients are positive and statistically distinguishable from chance. Starting with column (7), corresponding to our integrative survival index (*SI*) and in the absence of controls for alternative explanations of the uneven distribution in COVID-19 mortality during the first wave of the epidemic in Europe, we find that a standard deviation increase in the pre-existing survival environment increases the COVID-19 death rate by 73% (95% CI: 53, 92). In column (8), we find that a standard deviation increase in the pre-existing survival environment increases the COVID-19 death rate by 23%, other variables held equal. For context, a standard deviation increase in the survival index, for example, is the equivalent of moving from Greece (*SI* = 1.28) to Spain (*SI* = 2.22). The estimate of *ρ* is reported for all models in Table 1 along with the associated modified Bhargava et al. Durbin-Watson and Baltagi-Wu locally best invariant (LBI) test statistics under the null hypothesis ρ=0 [17,18]. This null hypothesis is rejected across all models presented in Table 1, implying that the modeled AR(1) disturbance structure appropriately corrects the model estimated standard errors.

**Figure 4:**
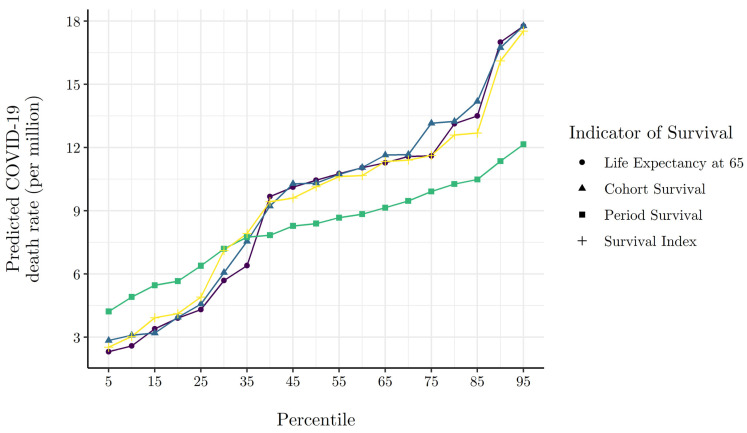
Model predicted cumulative COVID-19 death. Rate at various percentiles of survival environment indicators. NOTE: Model predicted cumulative death rates are generated via the Equation (12) model estimates presented in columns (1)–(4) of Table 1, where all other covariates are evaluated at their mean.

Figure 4 shows predicted COVID-19 death rates from Equation (12) across percentile scores of all indicators of the pre-existing elderly survival environment. We derive predicted COVID-19 death rates by fixing other model covariates pertaining to inbound international travelers, healthcare system capacity, the stringency of non-pharmaceutical interventions (NPI), population density, percent elderly, the confirmed case rate, and week of observation, set at their sample means. We observe high statistical agreement across indicators of the elderly survival environment. Results indicate that the expected COVID-19 death rate is 2.4× higher for countries at the 75th percentile of our survival index as compared to countries at the 25th percentile. Overall, Figure 4 implies that the rate of COVID-19 mortality increased linearly in pre-existing elderly survival over the first wave of the COVID-19 pandemic in Europe.

Table 2 reports coefficients from Equation (13), where measures of the elderly survival environment are divided categorically into terciles of low, medium, and high survival. Because our response variable is measured in natural log terms, reported coefficients have the interpretation of a semi-elasticity reflecting the percent difference in the COVID-19 death rate of a tercile of interest relative to the reference tercile I of low pre-existing elderly survival. Consistent with theoretical expectation, coefficients across all operational definitions of the elderly survival environment are positive and statistically significant. As with Table 1, we present coefficients with and without control variables for alternative explanations. Focusing on column (8), and all other things held equal, we find that countries in tercile II (medium survival environment) have an average COVID-19 death rate that is 85% higher (95% CI: 42, 171) than countries in tercile I (low survival environment). To obtain the marginal effects, in percentage terms, for the estimated coefficients in Table 2, one must perform the following transformation: $\left[\exp\left(\hat{\beta }\right)-1\right]\times 100$ [16]. Similarly, and relative to tercile I (low survival environment), countries in tercile III (high survival environment) have an average COVID-19 death rate that is 109% higher (95% CI: 51, 233). Results in Table 2 corroborate Table 1 and Figure 4, implying that, other things held equal, the COVID-19 death rate increased monotonically with pre-existing elderly survival over the first wave of the pandemic.

**Figure 5:**
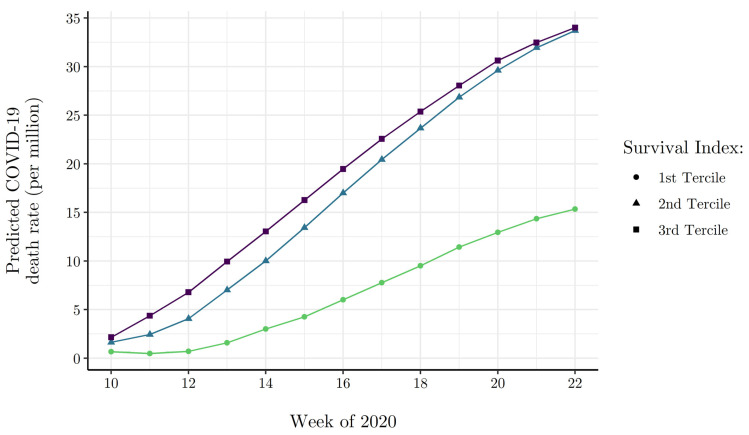
Model predicted cumulative COVID-19 death rate by week of Wave I for survival index terciles. NOTE: Model predicted cumulative death rates by week and survival index tercile are generated via Equation (14) model estimates, where all other covariates are evaluated at their mean.

Next, Figure 5 presents results from Equation (14), showing predicted cumulative COVID-19 death rates by Survival Index terciles over the course of the first wave of the European pandemic. Again, predictions are derived with all other model covariates fixed at their sample means. The results in Figure 5 corroborate the hypothesis that the pre-existing survival environment is positively associated with the COVID-19 death rate. By the 22nd week of the pandemic, the predicted COVID-19 death rate of high elderly survival countries stood at 34 deaths per million, about 2.2× higher than low survival environment countries.

## 5. Robustness

### 5.1. Excess Mortality

Given known inconsistencies in the reporting of COVID-19 deaths across countries—especially during the first wave of the pandemic—excess mortality can be used as a proxy for COVID-19 mortality that is potentially less biased by administrative differences that lead to under or over-reporting of COVID-19 deaths. We obtained weekly excess mortality *Z*-scores from EuroMOMO. Excess mortality *Z*-scores were accessed at https://www.euromomo.eu/graphsand-maps (accessed on 15 July 2020). Each observation in the EuroMOMO data reflects the within country deviation from central tendency, or baseline, in all-cause mortality for a given week. Because of the within-country differenced nature of EuroMOMO’s excess mortality measure, we estimate the following panel-corrected standard errors model

(15) $$\ln\left(EM_{jt}\right)=\alpha +\beta \ln\left(SEI_{j}\right)+\delta X_{j}+\gamma Z_{jt}+\epsilon_{jt}$$

where all terms carry from Equation (10), with the exception of our response variable *EM_jt_* which measures the cumulative excess mortality of country j in week t in standardized terms. $\epsilon_{jt}$ follows the same AR(1) disturbance structure defined in Equation (11).

Table 3 reports the estimated coefficients. As before, our interpretive focus is on column (4) corresponding to our integrative survival index. Because our survival index is measured in standardized terms, our coefficient of interest, β, has the interpretation of an elasticity, indicating that a standard deviation increase in the pre-existing survival environment is associated with a β percent change in excess mortality. Other things held equal, we find that a unit increase in our survival index, capturing the pre-existing elderly survival environment, is associated with a 5% increase (95% CI: 1, 9) in cumulative weekly excess mortality.

### 5.2. Age-Specific Mortality

The National Institute for Demographic Studies (INED) collects age- and sex-specific COVID-19 death data for select European countries. INED age- and sex-specific COVID-19 death data were accessed at https://dc-covid.site.ined.fr/en/data/ (accessed on 15 July 2020). We collapse age-specific COVID-19 deaths into common age intervals across the countries for which data are available and calculate corresponding pre-existing all-cause mortality rates using data from the Human Mortality Database (HMD). Age-specific COVID-19 death data is available for all age groups in England, France, Germany, Italy, the Netherlands, Spain, Sweden, and Ukraine. Denmark’s age-specific data is only available for those 60 years of age and older, Norway data is available for those 40 years of age and older, and Portugal’s data is top-coded at 80 years of age so that comparable data is only available for those 79 years of age and younger. Pre-existing age-specific all-cause mortality data are anchored on the most recently available common year of 2016. To test the statistical relationship between pre-existing all-cause mortality and the COVID-19 death rate, we estimate the following least squares model

(16) $$\ln\left(CoV_{ijt}\right)=\alpha +\beta \ln\left(ACDR_{ij}\right)+\delta_{i}+W_{t}+\varepsilon_{ijt}$$

where *CoV* is the COVID-19 death rate for age group *i* in country *j* at time *t*, *ACDR* is the pre-existing all-cause death rate (2016), $\delta_{i}$ is a vector of age-interval fixed effects, and *W_t_* is a vector of week fixed effects. *ε* is the model disturbance term. Our theoretical interest is in the estimate of *β,* which we expect to be negative, implying that, other things equal, a 1% increase in the age-specific all-cause death rate is associated with a *β* percent decrease in the associated age-specific COVID-19 death rate.

Table 4 reports the coefficient estimates for Equation (16). The model estimates presented in column (1) are generated in the absence of age-interval fixed effects. In columns (2) through (4) we introduce age-interval fixed effects. The inclusion of these variables results in a between-country estimate of what is purged of the confounding within-country effect of increasing risk of death across age intervals. According to our theoretical expectations, preexisting all-cause mortality rates are estimated to have a strong, negative association with the COVID-19 death rate across countries. The coefficient estimate of −1.439 (95% CI: −1.519, −1.360) in column (2) implies that, other things equal, a 1% increase in the pre-existing all-cause death rate is associated with an approximate 1.5% lower COVID-19 death rate, on average across age intervals.

In column (3) of the table we reduce the sample to intervals ≥ 60 years of age. The qualitative nature of the estimates is maintained, though the negative association between pre-existing all-cause mortality and the COVID-19 death rate appears to be stronger among the elderly. Specifically, a 1% increase in the pre-existing all-cause death rate is associated with an approximate 4.3% decrease in the COVID-19 death rate for those ≥ 60 years of age, other things equal. In column (4) we restrict the sample to intervals ≤ 49 years of age. Relative to column (3), which restricts to intervals ≥ 60 years of age, the coefficient estimate in column (4) intuitively deflates, reinforcing that the pre-existing death rate effect is stronger among the elderly and compatible with a longevity-frailty model.

## 6. Conclusions

In this paper we developed a simple model of longevity and accumulated health frailty to explain observed differences in COVID-19 mortality across the countries of Europe during the first wave of the pandemic. The model describes how heterogeneous survival environments across countries induce mortality selection processes that generate varying amounts of accumulated health frailty among elderly populations, accounting for the surprising statistical independence of age structure and COVID-19 death rates but also for the known increase in the risk of COVID-19 mortality with age. With respect to an exogenous health shock that disproportionately targets the relatively health frail, like SARS-CoV-2, our model implies that countries with a more favorable survival environment will have a larger susceptible (health frail) population and, therefore, a higher COVID-19 death rate.

To operationalize a country’s survival environment—a key parameter of our model—we collected data on pre-existing all-cause age-specific death rates (measured in both period and cohort-specific terms), life expectancy at age 65, and an index of survival that is integrative of these variables. We showed that our various indicators of the survival environment are correlated with more explicit measures of elderly frailty (see Appendix A Table A1 and Figure A1), serving as adequate proxies of relative differences in accumulated health frailty among the countries of Europe.

We then statistically linked cross-national variation in survival environments to the puzzling distribution of COVID-19 death rates in Europe at the end of spring, finding that a more favorable survival environment, as indicated by lower pre-existing all-cause mortality rates, is positively associated with COVID-19 death rates. Specifically, we found that a standard deviation increase in the pre-existing survival environment increased the COVID-19 death rate by 23%, other variables held equal. Coupled with analyses involving categorical measurement of the elderly survival environment, the COVID-19 death rate appeared to increase monotonically with pre-existing elderly survival over the first wave of the pandemic. Importantly, these relationships are robust to statistical control for the inbound international travelers, the capacity of a healthcare system to manage and survive infected persons, population density, percent elderly, epidemic intensity, and the timing and stringency of non-pharmaceutical interventions, among other factors.

We followed with analyses of cumulative excess mortality instead of the COVID-19 death rate. Results behaved similarly, implying that reporting differences across European countries is an unlikely source of our observed correlations between pre-existing survival environments and COVID-19 mortality. We closed analyses showing a strong negative association between age-specific COVID-19 death rates and pre-existing all-cause age-specific mortality rates for a subset of European countries with readily available data.

Our results are consistent with other known facts concerning COVID-19 mortality during the first wave of the pandemic. Specifically, end-of-spring data show that a large percentage of COVID-19 deaths occurred among frail populations in long-term care facilities—Belgium (53%), Denmark (34%), France (51%), Germany (36%), Hungary (19%), Ireland (60%), Norway (60%), Portugal (40%), and Sweden (45%). These data come from the International Long-Term Care Policy Network [19] and Sciensano, a public research institution that publishes very detailed epidemiological daily reports on COVID-19. In France, Spain, and Italy, among the hardest hit countries of Europe, about half to two-thirds of elderly require assistance for basic personal and household care activities. This fact educates that a sizeable percentage of European elderly could not easily socially distance in the first wave of the pandemic, compounding the risk of COVID-19 mortality.

In sum, our results support the hypothesis that variation in pre-existing frailty, stemming from differences in pre-existing survival environments, partially determined the striking differences in COVID-19 death risk observed during the first wave of the European pandemic. Our findings also provide general intuition as to how similarly novel causes of death might operate across countries via the heterogeneous accumulation of health frailty among their populations. It is interesting that some reports suggest that the aged population in developing countries show a lower COVID-19 mortality rate than seen in Europe, which would be predicted by our model due to the poor survival environment that the aged face in these countries [20].

## Figures and Tables

**Table 1:** Coefficients of interest for regression of *Ln* cumulative COVID-19 death rate on *Ln* survival environment indicators.

	(1)	(2)	(3)	(4)	(5)	(6)	(7)	(8)
Life Expectancy at 65 (LE)	12.73 *** (1.75)	4.17 ** (1.86)						
Cohort Survival (CS)			132.35 *** (17.88)	44.61 *** (16.92)				
Period Survival (PS)					176.67 *** (28.11)	45.19 * (24.18)		
Survival Index (SI)							0.73 *** (0.098)	0.23 *** (0.09)
Constant	−39.16 ***	−23.08	−572.02	−203.55 ***	−817.79 ***	−219.60 ***	−1.74 ***	−11.83 ***
	(5.16)	(5.90)	(77.07)	(72.48)	(129.87)	(111.62)	(0.20)	(2.51)
*ρ*ˆ	0.91	0.85	0.90	0.85	0.91	0.85	0.90	0.85
Bhargava et al. Durbin−Watson	0.39	0.36	0.37	0.36	0.39	0.36	0.37	0.36
Baltagi−Wu LBI	0.42	0.41	0.41	0.40	0.42	0.41	0.41	0.40
Observations	2670	2670	2492	2492	2670	2670	2492	2492
Countries	30	30	28	28	30	30	28	28
Country RE	Yes	Yes	Yes	Yes	Yes	Yes	Yes	Yes
Week FE	Yes	Yes	Yes	Yes	Yes	Yes	Yes	Yes
Controls	No	Yes	No	Yes	No	Yes	No	Yes

NOTE: Standard errors in parentheses. Levels of statistical significance are indicated by *** *p <* 0.01, ** *p <* 0.05, * *p <* 0.1. All models incorporate an AR(1) disturbance structure and the estimated *ρ* is reported along with the associated modified Bhargava et al. Durbin-Watson and Baltagi-Wu locally best invariant (LBI) test statistics, both of which have complicated distributions [17,18]. In all cases, we reject the null hypothesis of *ρ* = 0. Estimates are from Equation (12).

**Table 2:** Coefficients of interest for regression of *Ln* cumulative COVID-19 death rate on categorical survival environment indicators.

	(1)	(2)	(3)	(4)	(5)	(6)	(7)	(8)
LE Tercile II	1.80 *** (0.41)	0.65 ** (0.32)						
LE Tercile III	2.75 *** (0.41)	1.08 *** (0.39)						
CS Tercile II			1.80 *** (0.46)	0.52 (0.33)				
CS Tercile III			2.63 *** (0.46)	0.90 ** (0.39)				
PS Tercile II					1.22 *** (0.46)	0.40 (0.29)		
PS Tercile III					2.52 *** (0.46)	0.66 ** (0.34)		
SI Tercile II							1.85 *** (0.41)	0.84 *** (0.35)
SI Tercile III							2.86 *** (0.41)	1.09 *** (0.39)
Constant	−3.20 ***	−11.56 ***	−3.16 ***	17.18 ***	−2.93 ***	−11.40 ***	−3.25 ***	−13.44 ***
	(0.31)	(2.41)	(0.34)	(3.07)	(0.35)	(2.52)	(0.31)	(2.45)
*ρ*ˆ	0.91	0.85	0.90	0.90	0.91	0.85	0.90	0.85
Bhargava et al. Durbin−Watson	0.39	0.36	0.37	0.32	0.39	0.36	0.37	0.36
Baltagi−Wu LBI	0.42	0.41	0.41	0.35	0.42	0.41	0.41	0.40
Observations	2670	2670	2492	2492	2670	2670	2492	2492
Countries	30	30	28	28	30	30	28	28
Country RE	Yes	Yes	Yes	Yes	Yes	Yes	Yes	Yes
Week FE	Yes	Yes	Yes	Yes	Yes	Yes	Yes	Yes
Controls	No	Yes	No	Yes	No	Yes	No	Yes

NOTE: Standard errors in parentheses. Levels of statistical significance are indicated by *** *p <* 0.01, ** *p <* 0.05. For each column (1)–(4), the estimated coefficients are relative to the reference group, which is tercile I for each survival environment indicator. All models incorporate an AR(1) disturbance structure and the estimated *ρ* is reported along with the associated modified Bhargava et al. Durbin-Watson and Baltagi-Wu locally best invariant (LBI) test statistics, both of which have complicated distributions [17,18]. In all cases we reject the null hypothesis of *ρ* = 0. To obtain the marginal effects, in percentage terms, for the estimated coefficients above, one must perform the following transformation: $\left[\exp\left(\hat{\beta }\right)-1\right]\times 100$ [16]. Estimates are from Equation (13). LE is Life Expectancy at 65, CS is Cohort Survival, PS Period Survival, and SI is Survival Index.

**Table 3:** Coefficients of interest for regression of *Ln* cumulative excess mortality on *Ln* survival environment indicators.

Title	(1)	(2)	(3)	(4)
Life Expectancy at 65 (LE)	2.89 *** (0.84)			
Cohort Survival (CS)		28.56 *** (9.33)		
Period Survival (PS)			13.09 (9.24)	
Survival Index (SI)				0.05 ** (0.02)
Constant	−10.38 ***	−126.09 ***	−63.56	−3.60 ***
	(4.23)	(40.73)	(42.77)	(1.18)
*ρ*ˆ	0.92	0.92	0.91	0.93
Observations	247	234	247	234
R−squared	0.88	0.85	0.88	0.85
Countries	19	18	19	18
Week FE	Yes	Yes	Yes	Yes
Controls	Yes	Yes	Yes	Yes

NOTE: Standard errors in parentheses. Levels of statistical significance are indicated by *** *p <* 0.01, ** *p <* 0.05. All models incorporate an AR(1) disturbance structure and the estimated *ρ* is reported. Estimates are from Equation (15).

**Table 4:** Regression of *Ln* cumulative age-specific COVID-19 death rates on *Ln* pre-existing all-cause age-specific death rates.

	(1)	(2)	(3)	(4)
All−Cause Death Rate (2016)	1.112 ***	−1.439 ***	−4.313 ***	−0.653 ***
	(0.010)	(0.041)	(0.095)	(0.034)
Age 10–19		−0.758 ***		−0.169 **
		(0.104)		(0.072)
Age 20–29		1.664 ***		1.600 ***
		(0.089)		(0.061)
Age 30–39		3.595 ***		3.001 ***
		(0.093)		(0.065)
Age 40–49		5.570 ***		4.364 ***
		(0.106)		(0.078)
Age 50–59		8.101 *** (0.132)		
Age 60–69		10.408 *** (0.160)		
Age 70–79		12.831 ***	5.009 ***	
		(0.192)	(0.104)	
Age 80–89		15.571 ***	10.979 ***	
		(0.234)	(0.201)	
Age ≥ 90		17.749 ***	16.161 ***	
		(0.274)	(0.297)	
Constant	4.843 ***	−17.480 ***	−20.587 ***	−10.379 ***
	(0.501)	(0.487)	(0.649)	(0.581)
Observations	4697	4697	2335	1820
R−squared	0.738	0.869	0.710	0.764
Week FE	Yes	Yes	Yes	Yes
Age Interval FE	No	Yes	Yes	Yes

NOTE: Standard errors in parentheses. Levels of statistical significance are indicated by *** *p <* 0.01, ** *p <* 0.05. For columns (2) and (4) the reference age group is 0–9. For column (3) the reference age group is 60–69. Estimates are from Equation (16).

## Data Availability

Available from authors on request.

## Appendix A

**Table A1:** Survival and elderly care indexes by country.

Country	Survival Index	Cohort Survival	Period Survival	Life Expectancy at 65	Elderly Care Index	% ≥ 65 Disabled Need Help	% ≥ 65 Need Help Personal Activities	% ≥ 65 Need Help House Activities
France	2.557	1.522	1.590	1.404	2.270	1.400	0.678	1.839
Spain	2.220	1.271	1.410	1.249	2.258	1.496	1.266	1.149
Italy	1.975	1.172	1.208	1.125	2.208	1.816	0.810	1.185
United Kingdom	1.259	0.778	1.004	0.476	1.115	0.746	0.611	0.575
Norway	1.040	0.519	0.558	0.803	−1.486	−0.890	−1.076	−0.613
Luxembourg	0.978	0.192	0.744	0.839	−1.144	−0.362	−1.217	−0.418
Belgium	0.969	0.574	0.608	0.571	1.394	0.623	0.310	1.470
Sweden	0.951	0.578	0.402	0.744	−0.415	−0.528	−0.219	0.029
Denmark	0.805	0.370	0.843	0.256	−0.449	−0.785	0.464	−0.437
Iceland	0.776	0.970	−0.015	0.458	−1.525	−0.944	−0.905	−0.794
Austria	0.762	0.489	0.323	0.583	−1.514	−0.814	−0.840	−0.967
Finland	0.468	0.372	−0.024	0.536	−2.158	−1.460	−0.949	−1.322
Netherlands	0.416	0.440	−0.071	0.423	−1.114	−0.873	−0.822	−0.239

NOTE: This table presents standardized scores for three indicators of survival environment and the “Survival Index” alongside three measures of elderly frailty and the “Elderly Care Index” for 13 similarly economically developed countries. Survival environment indicator data are from the Human Mortality Database (HMD) (https://www.mortality.org/ (accessed on 15 July 2020)) and elderly frailty indicator data are from European Commission Eurostat database (https://ec.europa.eu/eurostat/data/database (accessed on 15 July 2020)).
